# Survival and DNA Damage in Plant Seeds Exposed for 558 and 682 Days outside the International Space Station

**DOI:** 10.1089/ast.2015.1457

**Published:** 2017-03-01

**Authors:** David Tepfer, Sydney Leach

**Affiliations:** ^1^ PESAC, Institut National de la Recherche Agronomique, Versailles, France.; ^2^ LERMA, Observatoire de Paris, PSL Research University, CNRS, Sorbonne Universités, UPMC Univ. Paris 06, Meudon, France.

## Abstract

For life to survive outside the biosphere, it must be protected from UV light and other radiation by exterior shielding or through sufficient inherent resistance to survive without protection. We tested the plausibility of inherent resistance in plant seeds, reporting in a previous paper that *Arabidopsis thaliana* and tobacco (*Nicotiana tabacum*) seeds exposed for 558 days outside the International Space Station (ISS) germinated and developed into fertile plants after return to Earth. We have now measured structural genetic damage in tobacco seeds from this EXPOSE-E experiment by quantitatively amplifying a segment of an antibiotic resistance gene, *nptII*, inserted into the chloroplast genome. We also assessed the survival of the antibiotic resistance encoded by *nptII,* using marker rescue in a soil bacterium. Chloroplast DNA damage occurred, but morphological mutants were not detected among the survivors. In a second, longer mission (EXPOSE-R), a nearly lethal exposure was received by Arabidopsis seeds. Comparison between a ground simulation, lacking UV_<200nm_, and fully exposed seeds in space indicated severe damage from these short wavelengths and again suggested that DNA degradation was not limiting seed survival. To test UV resistance in long-lived, larger seeds, we exposed Arabidopsis, tobacco, and morning glory seeds in the laboratory to doses of UV_254nm_, ranging as high as 2420 MJ m^−2^. Morning glory seeds resisted this maximum dose, which killed tobacco and Arabidopsis. We thus confirm that a naked plant seed could survive UV exposures during direct transfer from Mars to Earth and suggest that seeds with a more protective seed coat (*e.g.,* morning glory) should survive much longer space travel. Key Words: UV light—Flavonoids—Sinapate—DNA degradation—Arabidopsis—Tobacco—Seeds—Space—International Space Station—EXPOSE-E—EXPOSE-R. Astrobiology 17, 205–215.

## 1. Introduction

In an effort to explore the hypothesis that life could survive outside the biosphere, experiments have been performed on Earth in simulators and directly in space on rockets and satellites with organisms known to resist extreme environments on Earth. We proposed that plant seeds be considered model space travelers (Tepfer and Leach, 2006), because plant seeds inherently tolerate temperature extremes, vacuum, and desiccation; they accumulate potentially protective, UV-absorbing substances, particularly in their seed coats (Shirley *et al.,* 1995; Sheahan, 1996; Lepiniec *et al.,* 2006). Furthermore, their genomes are redundant, carrying multiple versions of essential genetic information. These attributes could contribute to overcoming damage accumulated during space travel and allow germination in a favorable environment. Their cells also could provide the macromolecular ingredients (*e.g.,* nucleic acids, proteins, ribosomes, and membranes) for a rapid renaissance of life, if the seeds themselves were killed (Tepfer and Leach, 2006). Seeds can also harbor bacteria and fungi capable of living autonomously (Clay and Schardl, 2002). Seeds are thus model organisms for testing the plausibility of the transfer of life through space (Tepfer, 2008).

Two long-term exposures have taken place on the outside of the International Space Station (ISS). In the EXPOSE-E mission, bacteria, lichens, communities of phototrophs, organic molecules, and seeds were exposed for 558 days, receiving 740 MJ m^−2^ of UV_110–400nm_ (Rabbow *et al.,* 2009). A second and longer mission to the ISS (EXPOSE-R) provided a higher exposure (1030 MJ m^−2^ of UV_110–400nm_) and included viruses, microorganisms and their spores, organic molecules, eggs of crustacean and cryptobiotic larvae, and seeds (Rabbow *et al.,* 2015). UV light was the dominant lethal factor for all species. Except for seeds, the rare cases of survival after exposure to full space conditions, including UV light, could be attributed to shadowing by killed surface organisms or through sample compartment edge effects. Seeds were in monolayers, exposed to space UV light, radiation, vacuum, and temperature changes. To avoid edge effects, seeds were only taken from a central zone of each seed monolayer, which was defined by germination studies where seeds were assayed sequentially, starting in the center of the monolayer. This safe zone was confirmed by modeling exposure loss from shadowing due to compartment edge effects and nearby objects (Tepfer *et al.,* 2012).

**FIG. 1.**
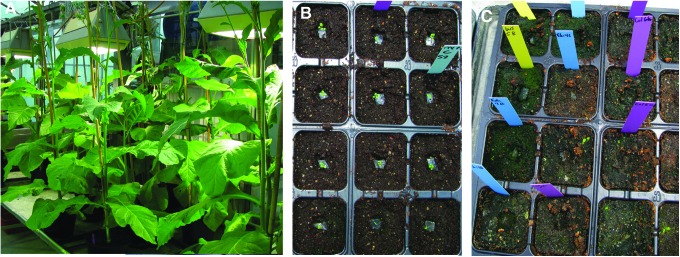
Whole plants from exposed seeds. (**A**) Mature survivors from the PT transgenic tobacco line from EXPOSE-E, showing unaltered growth and morphology. (**B**) Arabidopsis Columbia survivors from the S2 layer from EXPOSE-R, 1 week after transfer to soil in blocks of agar, showing adaptation to greenhouse conditions. (**C**) Remains of the 11 survivors from the S1 layer, 6 weeks after transfer to soil. (Green spots are due to the growth of algae in Perlite fragments of potting substrate.)

In the EXPOSE-E SEEDS experiment, we exposed Arabidopsis and tobacco seeds, purified DNA, and chemical samples of the flavonoid UV screens found in seed coats. Seed survival was assessed after return to Earth through germination tests *in vitro* and greenhouse studies, giving overall survival rates of 23% in wild type Arabidopsis and tobacco seeds. The surviving plants were fertile, albeit sometimes impaired in growth and seed production. They and their progeny showed no morphological abnormalities (Fig. 1A). Slow growth and reduced fertility essentially disappeared in the first sexual generation. Growth reduction was less common in tobacco than in Arabidopsis, which we attributed to the more redundant genome and developed endosperm in tobacco. We concluded that the loss of survival was likely not due to irreparable DNA damage but rather to dysfunction in cellular structures necessary for growth and development, for example, ribosomes, after the seed imbibed water prior to germination. The role of flavonoid and (to a lesser extent) sinapate ester UV shields was evident in the loss of viability in Arabidopsis mutants deficient in producing these compounds. Exposure of pure flavonoid samples caused loss of chemical integrity while maintaining the ability to absorb UV at DNA absorption maxima. Unprotected DNA was not completely degraded by this exposure, since a 110-base fragment of the model gene used, *nptII*, was amplified by polymerase chain reaction (PCR), but full copies were not (Tepfer *et al.,* 2012; see also Thiel *et al.,* 2014).

Intrigued by the lack of morphological abnormalities in Arabidopsis and tobacco survivors from EXPOSE-E, we examined chloroplast DNA from dry tobacco seeds from EXPOSE-E, using quantitative polymerase chain reaction (qPCR) to measure the physical integrity of the *nptII* gene. A complementary biological test, marker rescue in *Acinetobacter baylai* (a soil bacterium), served to assess the functional integrity of *nptII* after exposure, using the gene's kanamycin resistance function. In addition to genetic defects, exposure to space was expected to impair physiological functions, which must be overcome for seeds to germinate. As in EXPOSE-E, germination tests were used to measure the general viability of Arabidopsis seeds from EXPOSE-R and thus to estimate a survival endpoint in unshielded, low Earth orbit. Finally, using laboratory exposures to UV_254nm_, we compared the UV resistance of morning glory seeds, which are long-lived in nature (Brown and Porter, 1942), to that of Arabidopsis and tobacco, which are short-lived. Finally, we discuss the genetics and biochemistry of the liabilities of space travel and the feasibility of improving space extremophilia in seeds.

## 2. Methods

In the first mission (EXPOSE-E), Arabidopsis and tobacco seeds were exposed on the outside of the European Columbus module, which is attached to the American part of the ISS. They were held in monolayers behind magnesium fluoride (MgF_2_) windows, which transmit UV_>110nm_ (Tepfer *et al.,* 2012). The second mission (EXPOSE-R) was restricted to Arabidopsis seeds, and the sample holders were redesigned with round instead of square windows. In both missions, the top, fully exposed layer was designated S1 in space and G1 in the ground simulation. The dark layers beneath were labeled S2 and G2.

### 2.1. Biological materials

Tobacco was chosen for small seed size (*ca.* 700 μm in length) and the availability of the transgenic line, Havana PT (pTNH32-70-2, a gift from P. Maliga), carrying a functional kanamycin antibiotic resistance gene (*nptII*) in the chloroplasts (Carrer *et al.,* 1993). Chloroplasts, which are derived from ancient bacteria, are photosynthetic, intracellular organelles with simple genomes; they are numerous in plant cells, providing high copy numbers of the model gene. Tobacco is an allotetraploid, that is, a fusion of two different diploid genomes. It has a high level of genetic redundancy and a large genome (4.5 Gb) (Sierro *et al.,* 2013). Tobacco also has a developed endosperm, which surrounds and shields the embryo, compared to the vestigial endosperm in Arabidopsis. These characteristics could contribute to resistance to UV and cosmic radiation.

Arabidopsis was chosen for its very small seeds (*ca.* 500 μm in length) and the availability of two mutants deficient in two types of UV screen production. Two wild types (ecotypes) were tested: Columbia (Col-0) and Wassilewskija (Ws-2). The corresponding mutants were *fah1-2* (Nottingham Arabidopsis Stock Centre), which are defective in producing sinapate esters that protect against UV light (Chapple *et al.,* 1992), and *tt4-8*, which cannot make the flavonoid sunscreens (Li *et al.,* 1993). Aside from *fah1-2*, all other mutant and wild type Arabidopsis seeds were from the Versailles Arabidopsis Resource Centre. In contrast to tobacco, the Arabidopsis genome has a low level of genetic redundancy, a small genome (0.13 Gb) (The Arabidopsis Genome Initiative, 2000), and a vestigial endosperm.

Arabidopsis and tobacco seeds were produced in the greenhouse simultaneously for all genotypes of the same species. Morning glory (*Convolvulus arvensis*) seeds were collected in the wild near Versailles, France. Seeds were arranged in monolayers behind MgF_2_ windows, using a dissecting microscope and fine forceps. The same seed stocks served for EXPOSE-E and EXPOSE-R.

### 2.2. Seed removal and germination tests

These methods are described in more detail elsewhere (Tepfer *et al.,* 2012). Seeds were only taken from the center portion of each monolayer in order to avoid shadows caused by the edges of the sample wells. In EXPOSE-E, seeds were removed one by one from the monolayers, starting in the center and moving in a spiral outward. The estimated exposure falloff due to edge effects was <20%, and a germination gradient from the center to the edge of the sampled area was not detected. Therefore, in this report, seeds were removed in bulk from the center of the monolayer with a mild vacuum pulled on a 1 mL micropipette tip, which carried a sintered plastic plug in the wide extremity, keeping the seeds from being pulled into the vacuum line. After return to Earth, sample carriers and seeds were stored desiccated at 4°C.

Germination was tested *in vitro,* in Petri dishes containing medium that consisted of 0.8% agar (w/v) in deionized water. For both species in EXPOSE-R, 150 seeds from the center of each monolayer were sampled in three replicas of 50 seeds per Petri dish. Seeds from behind two dispersed windows were analyzed separately, except for the Ws seeds, which came from the single central sample well (in a total of nine wells, all covered by a single MgF_2_ window). Lab controls (L0) were stored at 4°C. Loaded Petri dishes were maintained at 8°C for 48 h in the dark to attain homogeneous imbibition before the start of germination at 22°C, under 16 h of 4 μE m^−2^ s^−1^ light from white compact fluorescent lamps (Philips Softone SW) and red LED lamps (Philips E27G50R). Germination was defined as root emergence, which is the endpoint of the germination process. It was monitored hourly during periods of rapid change, under 14 × magnification, and recorded on the back of the Petri dish, using a colored symbol for each time point. Samples were identified by numbers. Germinated seedlings were removed from Petri dishes and transferred to soil in the greenhouse in squares of agar, to avoid damage to the young roots (Fig. 1B).

The same methods were applied to seeds exposed to UV_254nm_ in the laboratory, except that all the seeds were pooled, since there was no shadowing due to the absence of edges and a stationary light source. Morning glory seeds do not germinate until the seed coat is broken, which was accomplished by using a rotating cutting disk on a Dremmel tool to nick the seed coat in the vicinity of the cotyledons, that is, away from the embryo, while holding the seed with forceps.

### 2.3. Space missions

The EXPOSE-E mission was launched by NASA on February 7, 2008 (flight STS-122) and returned on September 12, 2009 (flight STS-128 Cal). Exposure lasted 558 days, and sample de-integration took place on December 3, 2009. Preflight and postflight temperatures were ambient. Preflight seeds were stored in EXPOSE-E under nitrogen; postflight, they were maintained in ambient atmosphere. After de-integration, seeds were stored in the sample carrier and desiccated at 4°C.

EXPOSE-R was launched to the ISS on a Russian Progress spacecraft from Baikonur, Kazakhstan, on November 26, 2008, before the return of EXPOSE-E. (It was therefore not possible to take the results from EXPOSE-E into account in the design and execution of the EXPOSE-R experiments.) EXPOSE-R was mounted on a Russian Zvezda module, attached to the external URM-D platform by a Russian cosmonaut on March 11, 2009. The SEEDS experiment was in Tray 1, compartment 3. As in EXPOSE-E, our sample chambers were vented to space. Seeds in EXPOSE-R were subjected to external space conditions for 682 days, until January 21, 2011. Sample trays were brought back to Earth by the last Discovery shuttle flight, STS 133, on March 9, 2011. Total mission duration from launch to landing was 833 days. The complete mission was 950 days, from tray closure after sample integration to tray opening after return to Earth.

### 2.4. Conditions and exposures

Four UV sensors (photodiodes) were installed in each upper corner of the EXPOSE-E and EXPOSE-R facilities. UV radiation fluence was modeled by RedShift Design and Engineering BVBA, Belgium, including the calculation of shadowing effects caused by ISS geometry and attitude, as well as window transmission. Dosimetric data on cosmic radiation exposure was provided by experiment R3DR, using ionizing radiation spectrometers as detectors (Dachev *et al.,* 2015), and by thermoluminescence dosimeters (Berger *et al.,* 2015) located under the sample carriers in another tray.

Seeds in the ground simulation (G1) were exposed to UV_200–400nm_ from a solar simulator (SOL2000, Dr. Hönle GmbH SOL2000, Gräfelfing, Germany), over a period of 1 month at the German Aerospace Center (DLR), Cologne, Germany. A temperature plus vacuum simulation followed the UV exposure.

Seeds in space and in the ground simulation were held in monolayers against MgF_2_ by a steel plate resting on a coiled spring (EXPOSE-E) or by a hollow cylinder with a spring inside (EXPOSE-R). The individual, square MgF_2_ windows in EXPOSE-E were replaced in EXPOSE-R by one square MgF_2_ window that covered all nine samples. Sample wells in EXPOSE-R were round instead of square in EXPOSE-E. In both missions, sample holders were identical in space and in the ground simulation. EXPOSE-E and EXPOSE-R design was by Kayser-Threde GmbH (OHB System AG, Munich, Germany) and construction by RUAG Space, Zurich, Switzerland.

In addition to the above treatments, seeds were exposed to UV_254nm_ in the laboratory under a chemical fume hood with the sash open 8 cm on three sides, providing a continuous flow of air at ambient temperature in order to minimize temperature differences over the exposed surface. Seeds were spread in monolayers in shallow wells in two glass plates (11 × 5.6 cm, VWR International, France), with two ridges on the bottom surface to facilitate airflow. The sample wells had slightly concave bottoms and round cavities (Ø 15 mm). UV_254nm_ light was from a low-pressure, unfiltered, mercury lamp (VL-100G, Vilber Lourmat, France), placed 14 cm above the surface of the samples. It provided nearly uniform exposure, as detected at seed level by radiometers (VLX-254, VLX-312, and VLX-3W, Vilber Lourmat, France) equipped with sensors for 254, 312, and 356 nm. Besides UV_254nm_ light (90%), the lamp emitted in the UV_312nm_ (8%) and UV_365nm_ (2%). UV_254nm_ flux was measured weekly to account for exposure attenuation due to lamp ageing in the UV_254nm_ dose determinations. Trays were exchanged and rotated weekly to homogenize exposure. Samples exposed to lower doses and dark controls remained in the same glass trays as the exposed seeds during the entire experiment, but they were protected by glass microscope slides wrapped in aluminum foil when UV exposure was not required. Doses were 599, 871, 1120, and 2420 MJ m^−2^, corresponding to the 60-, 90-, 120-, and 291-day exposures.

### 2.5. DNA extraction, qPCR, and marker rescue

For each treatment (S1, S2, G1, and G2), the PT (chloroplast transgenic) dry tobacco seeds from the center, that is, tiers 1 and 2 (Tepfer *et al.,* 2012), of three monolayers on EXPOSE-E were pooled and extracted by first grinding in liquid N_2_ in the presence of wild type Havana tobacco seeds. These carrier seeds provided genetically neutral biomass to increase extraction efficiency to compensate for the small sample masses. Given the restricted surface area available on EXPOSE-E, DNA amounts were limited, constraining the marker rescue experiments, which require more DNA than qPCR. After grinding, DNA was extracted and purified with a Qiagen plant DNA extraction kit. Sample seed mass was determined on a Sartorius CP2-P microbalance (Göttingen, Germany).

We used qPCR to compare the relative integrity of a 110 base pair region (nucleotides 79–189) of the *nptII* gene, located between primers 5'AGACAATCGGCTGCTCTGAT and 5'CTCGTCCTGCAGTTCATTCA, copies of which had survived the full solar (S1) EXPOSE-E exposure (Tepfer *et al.,* 2012). Primers were designed by using Beacon Designer 4.0 software (Bio-Rad, USA). qPCR was performed in a CFX96 Touch real-time PCR detection system with the iQ SYBR Green Supermix (Bio-Rad), using the following steps for 39 cycles (after an initial treatment at 95°C for 15 min): 95°C for 10 s; 55°C for 30 s; 72°C for 30 s. Samples were in triplicate.

The relative biological activity of *nptII* in the DNA extracted from exposed and control tobacco seeds was estimated in *Acinetobacter baylyi* (strain BD413), which carried a defective copy of *nptII* on plasmid pMR7, in the form of a 10-base deletion starting at coding sequence position 5999 (de Vries and Wackernagel, 1998). A more detailed protocol is published elsewhere (Tepfer *et al.,* 2003). Briefly, foreign DNA assimilation by *A. baylyi* BD413 in liquid culture resulted in homologous recombination and replacement of the defective bacterial sequences by the chloroplast sequences, resulting in kanamycin resistance. The frequency of the marker rescue event, expressed in colony-forming units (CFU) or the number of kanamycin-resistant bacterial colonies, gave a relative and global estimate of the functional integrity of the incoming chloroplast DNA. Unlike qPCR, which copies DNA between precise points in the nucleotide sequence, marker rescue depends on homologous recombination that can occur throughout the homologous region. A preliminary experiment (not shown), using naked *nptII* DNA, produced by PCR, demonstrated a quantitative relationship between a range of increasing UV_254nm_ doses and a corresponding reduction in the frequency of rescue of the deletion in *nptII,* as measured by marker exchange.

DNA was quantified by A_260_ measurements with a Nanodrop 8000 UV-vis spectrophotometer (Thermo Scientific, USA) in multiple 1 μL aliquots from each DNA sample. DNA input was adjusted accordingly. Differences in the ratio between carrier and transgenic seeds, due to the impossibility of weighing exactly the same seed mass in each sample, were accounted for in the results.

### 2.6. UV_125–340nm_ absorption spectrometry

UV_125–340nm_ absorption was determined by using the ASTRID synchrotron facility at the University of Aarhus, Aarhus, Denmark. Methods are described in detail elsewhere (Zalar *et al.,* 2007b). Seed coats were prepared by rubbing dry Arabidopsis seeds between two layers of fine emery paper and recovering the seed coat fragments under a dissecting microscope with jeweler's forceps. Closely packed seed coats were attached to MgF_2_ discs by moistening with ultrapure water. Release of mucilage from the seed coats caused them to adhere after the water had evaporated. Salmon sperm DNA (Sigma-Aldrich) was purified as described (Tepfer *et al.,* 2012). The quercitrin and DNA absorption curves are from our previous work (Zalar *et al.,* 2007b), and the solar emission curve was from the work of Thuillier *et al.* (2004).

### 2.7. Statistical analysis

Analysis of variance (ANOVA) and the Student–Newman–Keuls multiple comparison were used to evaluate statistical differences, along with the graphing and statistics program, KaleidaGraph. *P* < 0.05 was considered significant.

## 3. Results

### 3.1. Experimental difficulties and uncertainties

Post-exposure sample analysis on Earth was complicated by limited sample (*e.g.,* an error in sample treatment could not be corrected by starting again in space), and only a few samples could be tested (particularly in EXPOSE-R). Achieving statistical significance from small surface areas required seed numbers only attainable with small seeds. Samples had to be repeated and dispersed on the exposed surface in order to mitigate local shadowing by nearby fixed objects, such as sample chamber edges, solar panels, other experiments (*e.g.,* installed on the EuTef platform in EXPOSE-E), and the Columbus module (EXPOSE-E) and docked spacecraft. These were accounted for in photon dose calculations through shadow mapping by RedShift (see Methods.) An onboard computer failure during EXPOSE-R resulted in the loss of 6 months of sensor data. Simulations served as a replacement (Rabbow *et al.,* 2015).

Cosmic radiation doses in the dark (S2) layer in space are rough estimates, since dosimeters were not included in our sample tray, and the dark measurements made in another tray were from behind the sample carriers, with windows, biological samples, and the backs of the carriers acting as shields. At this deep level, radiation was essentially from galactic cosmic rays with a high estimate of 368 ± 27 μGy per day or total doses of *ca.* 200 mGy for EXPOSE-E and (assuming similar conditions) *ca.* 300 mGy for EXPOSE-R. The actual dose received by seeds in the S2 layer was higher and qualitatively more diverse, because they were only shielded by the S1 layer, which in EXPOSE-R was covered by a common window and in EXPOSE-E covered by individual windows.

Postflight inspection of the windows from both missions revealed contamination by UV-absorbing substances, which were studied in detail for EXPOSE-R (Demets *et al.,* 2015). Window discoloration was not evident in the postflight windows that covered our samples, but our window was not included in the postflight transmission study. Neighboring AMINO windows lost UV transmission during exposure due to a colorless deposit, perhaps from rocket exhaust (Demets *et al.,* 2015). UV dose determinations were used to take into account transmission loss, but they are approximate, since the kinetics of window transmission change were not recorded. Instead, an average for the mission was applied, based on starting and ending data and the transmission spectrum of MgF_2_.

### 3.2. Chloroplast DNA damage in tobacco during EXPOSE-E

The lack of mutant phenotypes in the survivors (and in their progeny) of full space exposure in EXPOSE-E (Fig. 1A) raised the question of whether DNA damage had occurred. Quantification of chloroplast DNA damage in tobacco seeds from EXPOSE-E, using a bacterial kanamycin resistance gene (*nptII*) inserted into the chloroplast genome (Carrer *et al.,* 1993), revealed structural and functional damage correlated with exposure to UV light.

**FIG. 2.**
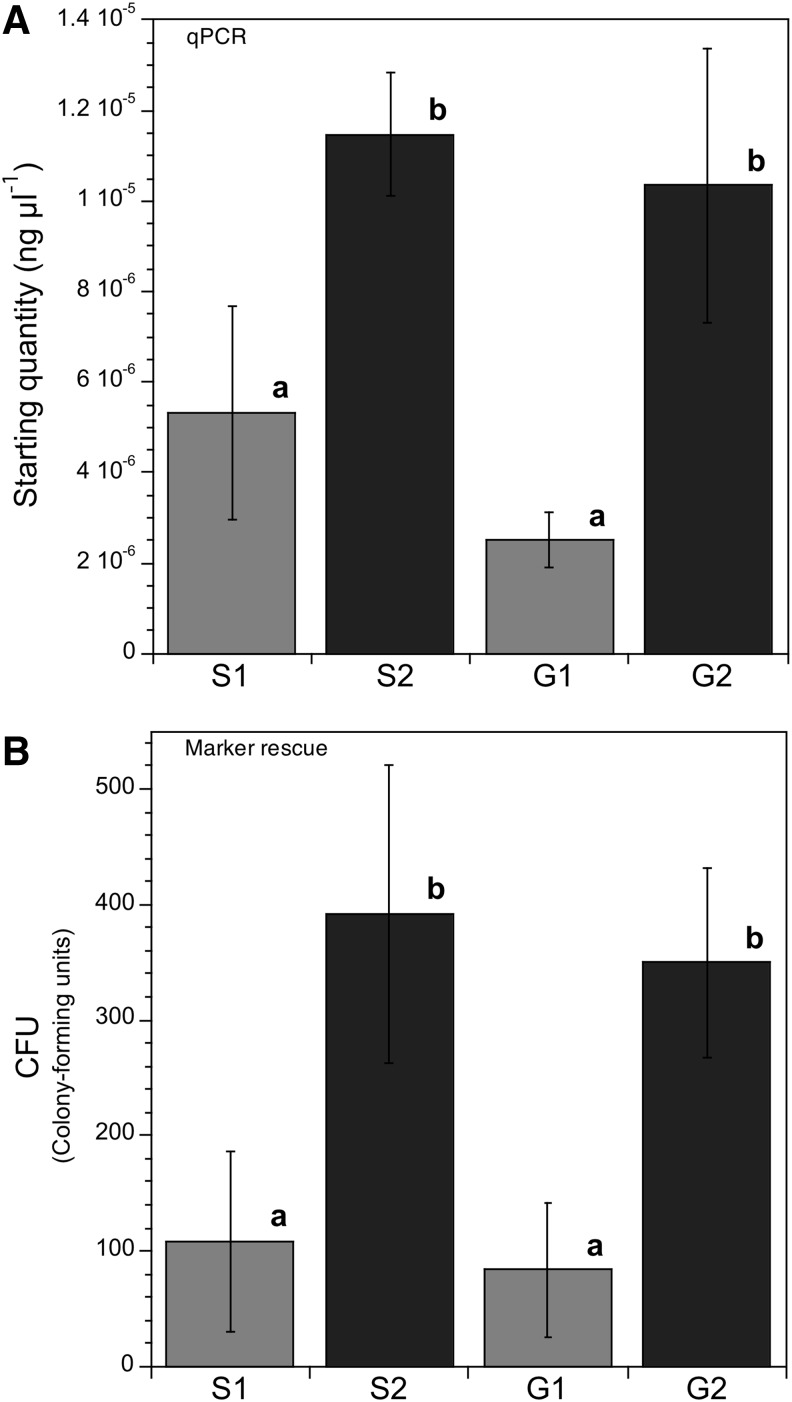
Integrity of chloroplast DNA from the PT transgenic line, as measured by qPCR and marker rescue. (**A**) qPCR (structural integrity) showing two statistically different groups, composed of UV-irradiated seeds (S1 and G1) vs. seeds from dark layers (S2 and G2). The G1 layer was not exposed to UV_<200nm_ and cosmic radiation. (**B**) A similar result obtained by marker rescue (functional integrity). Statistically similar results are designated by the same lowercase letter. *n* = 3 for qPCR; *n* = 4 for marker rescue; *P* < 0.05 was considered significant.

The sensitivity of qPCR and the high number of chloroplasts in tobacco seeds allowed a relative measure of DNA structural integrity, as defined by the number of amplifiable copies of a 110 base pair segment from the *nptII* gene copies in the global DNA extract (see Methods). The S1 seeds (exposed to full space conditions, including UV_110–400nm_) and the G1 seeds (exposed on Earth to simulated space conditions, including UV_200–400nm_, but lacking UV_<200nm_ and cosmic radiation) contained similarly low amounts of amplifiable chloroplast DNA (Fig. 2A). Seeds from the dark layer in space (S2) and in the ground simulation (G2) contained high amounts of amplifiable *nptII* DNA, compared to seeds from the corresponding fully exposed layers (S1 and G1). There was 2.1 times more amplification in the S2 than in the S1 DNA and 4.1 times more amplification in the G2 than the G1 DNA. Thus, DNA was damaged to a similar extent in seeds exposed in space and on the ground, even though the seeds in the ground simulation were not exposed to UV_<200nm_.

To complement the qPCR measures, we used the soil bacterium *A. baylyi* (strain BD 413) to assess the relative functional survival of *nptII* in the four DNA samples. This test relied on recombination between incoming DNA from the seed extract and target plasmid DNA in *A. baylyi* line BD413, which carried a mutated *nptII* (de Vries and Wackernagel, 1998). The sites of recombination cannot be known, and they vary with each rescue event. The foreign seed DNA can contain defects (*e.g.,* cross-linking, chain breakage, and frame shifts) that silence the gene and inhibit rescue of the deletion. (Frame shifts are not detected by qPCR.) The results from marker rescue are thus relative measures of the global functionality of the incoming seed DNA. Despite these differences, the marker rescue test of *nptII* function corroborated the structural study using qPCR: the two fully exposed layers were similarly low, and the two dark layers were similarly high (Fig. 2B). Comparison of the top layers that had received UV to the dark layers below them showed 3.6 times better DNA survival in S2 than in S1 and 4.2 times better survival in G2 than in G1.

### 3.3. A nearly lethal exposure to space in Arabidopsis during EXPOSE-R

**FIG. 3.**
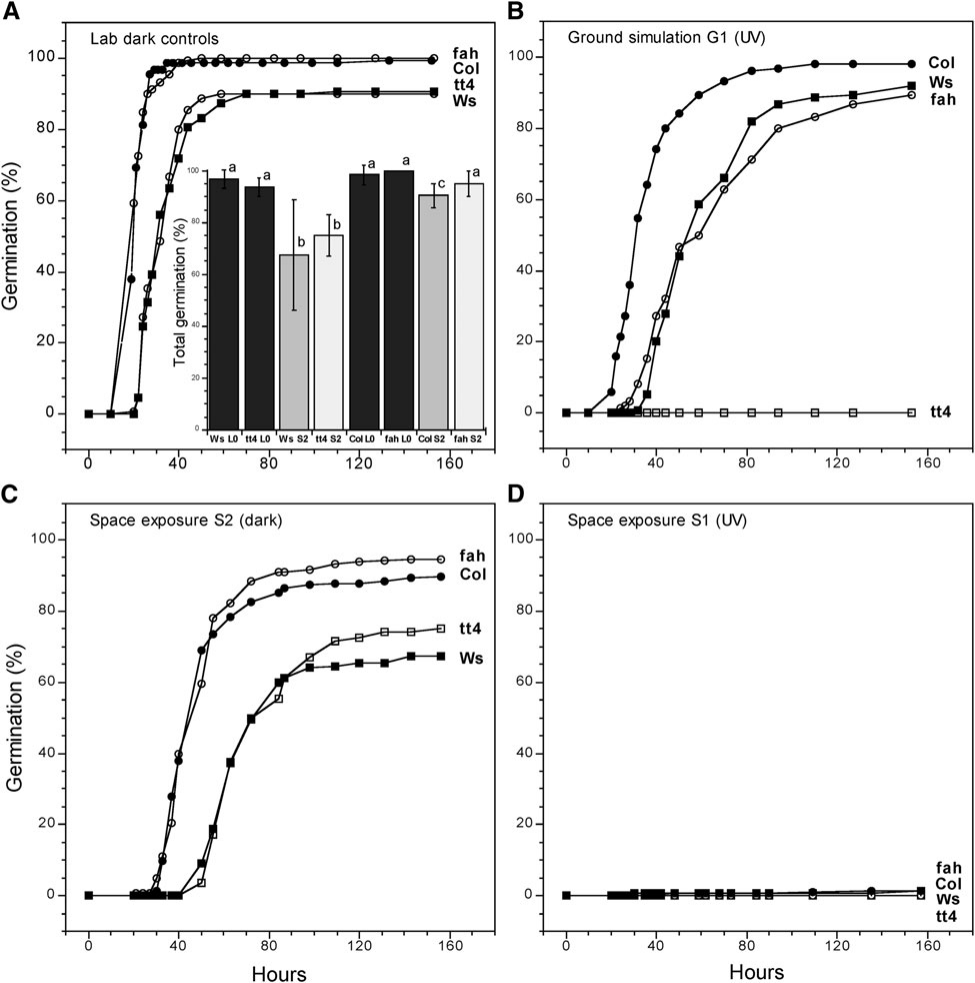
Germination of exposed and control seeds after return to Earth in EXPOSE-R. (**A**) Dark controls maintained in the laboratory. (**B**) Seeds from the ground simulation (G1) exposed to UV_200–400nm_, space vacuum and temperature. (**C**) Seeds exposed in space (S2) to space cosmic radiation (*ca.* 75–85% of S1), space vacuum and temperature, but not to UV. (**D**) Seeds exposed to full space conditions behind MgF_2_ windows, including UV_110–400nm_. The inset to (A) compares the germination endpoint values in (A) and (C). Black bars, dark lab controls; gray bars, Ws and Columbia wild types; light gray bars, the corresponding *tt4-8* (flavonoid-lacking) and *fah1-2* (sinapate-lacking) mutants. Each point is the mean of results from two dispersed sample wells (*n* = 150 for each sample well), with the exception of Ws, where only one sample well was sampled. Statistically similar results are designated by the same lowercase letter. *P* < 0.05 was considered significant.

Germination is the first step in the development of a plant from the embryo carried by a seed. Its timing and the final level of germination reached in a seed population give global measures of seed health. Postflight seed germination in Arabidopsis wild types from EXPOSE-R was a maximum of 3% in the *fah1-2* sample (Fig. 3D). With the exception of the *tt4* mutants, the others germinated at just over 1%, and the *tt4* mutants (lacking flavonoids) did not germinate at all. Only 11 seeds formed plants, and none of these survived transfer to the soil (Fig. 1B, 1C). A nearly lethal dose was thus defined for both the Ws and Columbia Arabidopsis ecotypes.

### 3.4. Lethality of UV_<200nm_ and cosmic radiation in Arabidopsis in EXPOSE-R revealed in germination tests

Ground simulations mimicked the conditions in space with the use of identical samples and sample holders. However, cosmic radiation was attenuated on Earth, and UV_<200nm_ light was absent in the ground G1 exposures. Comparison of germination in seeds from the G1 and S1 layers (Fig. 3B, 3D) thus gave a measure of the combined lethality of cosmic radiation and the UV_110–200nm_ in low-orbit space. This analysis had been attempted in EXPOSE-E, but not reported, because of an unexplained artifact in the G1 layer: seeds of both species were blackened in uneven, mottled patterns. In addition, the total UV exposure in EXPOSE-E was lower in G1 than in S1 (Table 1). Seed blackening did not occur in EXPOSE-R, so the S1 and G1 exposures could be compared.

**Table 1.** Materials and Exposure to Space Conditions

	*EXPOSE-E*	*EXPOSE-R*	*UV_254nm_ in lab*
Species	Arabidopsis, tobacco	Arabidopsis	Arabidopsis, tobacco, morning glory
Mutants			
Arabidopsis	Flavonoid or sinapate deficient	Flavonoid or sinapate deficient	None
Tobacco	*nptII* chloroplast insertion		
Dark controls	*In situ* (S2), lab (G2)	*In situ* (S2), lab (G2)	*In situ*
Biological parameters measured	Germination, plant development, fertility; integrity of chloroplast DNA	Germination	Germination
Chemicals exposed	*nptII* DNA, flavonoids	None	None
Exposures	558 days	682 days	60–291 days
UV radiation			2420 MJ m^−2^ UV_254nm_^c^ (maximum dose)
Space (S1)^a^	740 MJ m^−2^ UV_110–400nm_	1030 MJ m^−2^ UV_110–400nm_	
Space (S2)	None	None	
Ground (G1)^b^	580 MJ m^−2^ UV_200–400nm_	1130 MJ m^−2^ UV_200–400nm_	
Ground (G2)	None	None	
Cosmic radiation			
Space (S1)^d^	296 mGy	461 mGy	1–3 μGy day^−1^
Space (S2)^e^	*ca.* 250–300 mGy	*ca.* 350–400 mGy	1–3 μGy day^−1^
Ground (G1)^f^	1–3 μGy day^−1^	1–3 μGy day^−1^	
Ground (G2)^g^	1–3 μGy day^−1^	1–3 μGy day^−1^	
Temperature^h^	−25°C to +61°C	−25°C to +50°C	Ambient
Pressure	10^−4^ to 10^−7^ Pa	10^−4^ to 10^−7^ Pa	Ambient

^a^ S1: Space 1, the top layer, exposed to UV_110–400nm_ in space.

^b^ G1: Ground 1, the top layer, exposed to UV_200–400nm_ on the ground.

^c^ Maximum of four doses: 60, 87, 1132, and 2420 MJ m^−2^.

^d^ Galactic cosmic rays plus solar wind.

^e^ Cosmic radiation in the S2 layers was attenuated by the S1 samples and sample holders in both missions. The given doses are rough estimates based on dosimeters beneath the carriers in other trays and shielding estimates from the S1 layer.

^f^ Galactic cosmic rays.

^g^ Galactic cosmic rays.

^h^ EXPOSE-R included 285 freeze-thaw cycles, including 11 that were longer than the 90 min orbital period.

As expected after a heavy dose of UV_200–400nm_ in EXPOSE-R (Table 1), the G1 *tt4-8* (flavonoid minus) seeds did not germinate (Fig. 3B). In contrast, the Ws, Columbia, and *fah1-2* seeds showed robust (albeit slower) germination, arriving at or near the laboratory dark controls. Furthermore, in the G1 layer (Fig. 3B), the sinapate ester-deficient *fah1-2* mutant lost 11% viability (*P* = 0.0038), compared to the laboratory dark control (Fig. 3A), which is in keeping with the previously observed, less important role of the sinapate esters in the EXPOSE-E S1 seeds (Tepfer *et al.,* 2012). Flavonoids were thus essential to resistance against UV_200–400nm_, but they did not protect against full exposure to space, including UV_110–400nm_ and cosmic radiation, as seen in the near failure of germination in all seeds in the S1 layer (Fig. 3D). However, flavonoids protected efficiently in the G1 layer (Fig. 3B), when UV_110–200nm_ and cosmic radiation were absent, but space vacuum and temperature variation present, as seen in the total loss of viability in the *tt4-8* mutant and the robust survival of the Ws and (in particular) the Columbia wild types (Fig. 3B).

In the S2 seeds, which received no UV, germination in Ws and *tt4-8* was reduced and slower than in the corresponding laboratory dark controls (Fig. 3A, 3C). A lesser reduction occurred in the wild type Col seeds, but the *fah1-2* seeds were not significantly different from the laboratory dark controls (*P* = 0.746). The inset to Fig. 3A gives the endpoints for each seed type in Fig. 3A and 3C, with statistically similar samples designated by the same lowercase letter. The S2 seeds were subjected to the low-orbit cosmic radiation that was lacking in the ground simulation, albeit attenuated by the S1 layer (see Methods and Discussion).

### 3.5. Resistance of morning glory seeds to UV_254nm_ doses that were lethal to Arabidopsis and tobacco (laboratory experiments)

**FIG. 4.**
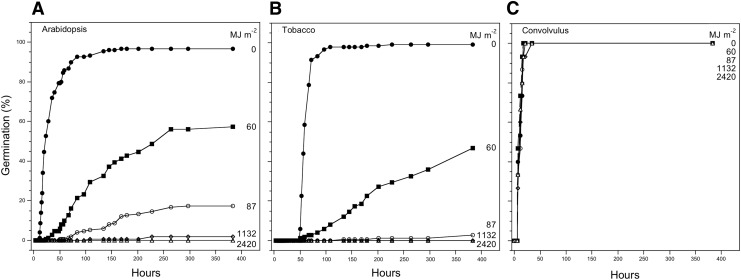
Exposure of Arabidopsis, tobacco, and morning glory seeds to UV_254nm_ in the laboratory for doses of 0, 60, 87, 1132, and 2420 MJ m^−2^. (**A**) Arabidopsis (Columbia). (**B**) Tobacco (Havana PT). (**C**) Morning glory (*Convolvulus arvensis*). *n* = 150 in three replicas of 50 seeds each (Arabidopsis); *n* = 15 (morning glory).

Sample number and size constraints restricted experimentation in space to small seeds, but small seeds are generally not capable of long-term survival in the soil. We thus extended our search for UV resistance to morning glory seeds, for their larger size, tougher seed coats, and longevity in the soil (Brown and Porter, 1942). Since further experiments in space were not possible, seeds were exposed in the laboratory to a series of UV_254nm_ doses ranging up to 2420 MJ m^−2^, which is 2.35 times the UV_110–400nm_ dose in the EXPOSE-R experiment. (See Methods for contaminating wavelengths.) UV_254nm_ concentrates energy in a crucial part of the spectrum, since DNA and the flavonoid sunscreens have absorption peaks near 260 nm. (Zalar *et al.,* 2007a). Germination in Arabidopsis and tobacco seeds slowed and diminished as a function of dose (Fig. 4A, 4B) but with most of the loss of viability occurring below 87 MJ m^−2^. The maximum exposure of 2420 MJ m^−2^ was completely lethal to Arabidopsis and tobacco seeds, but all the morning glory seeds germinated normally and produced viable plants at all doses (Fig. 4C).

### 3.6. UV_100–340nm_ absorption spectroscopy through Arabidopsis seed coats

**FIG. 5.**
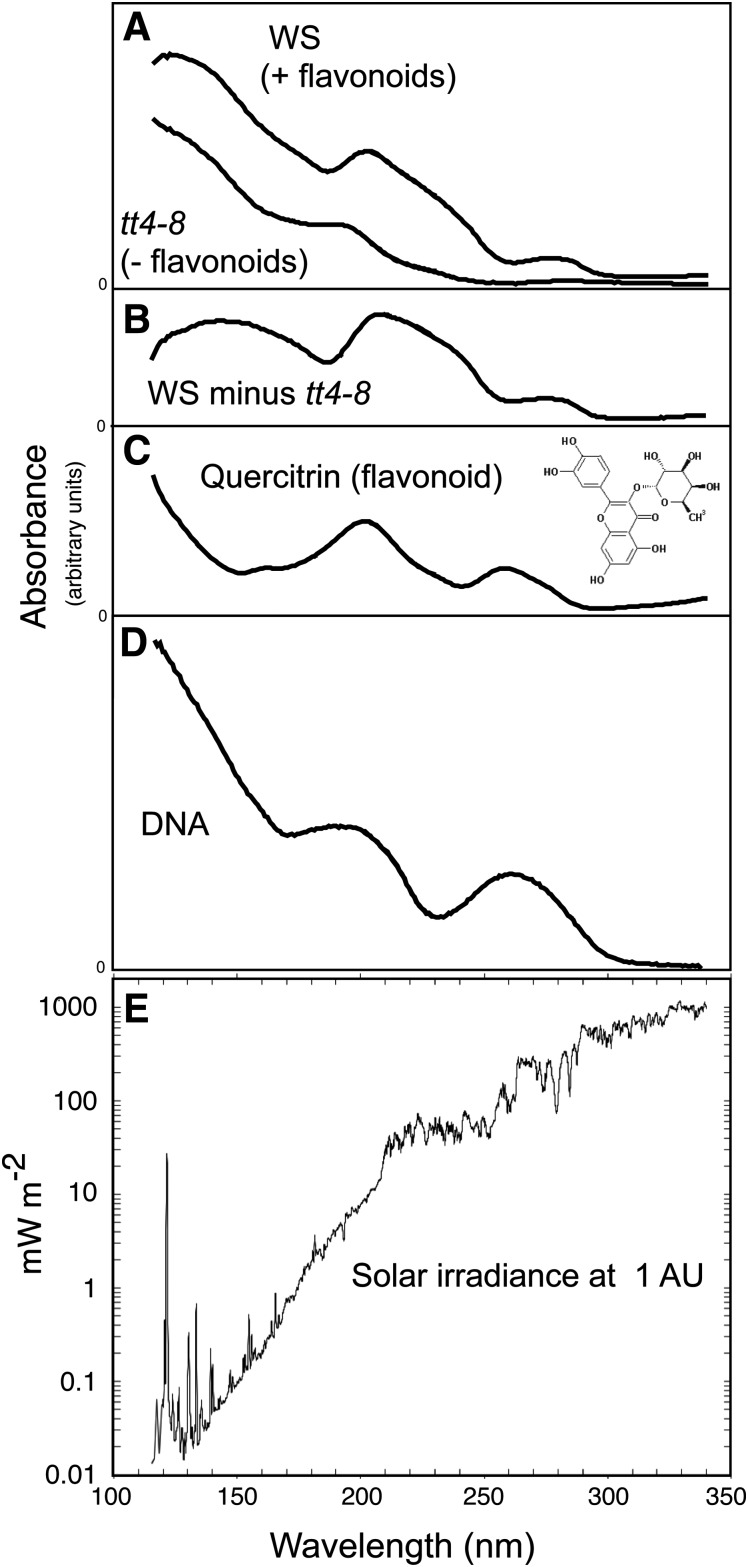
UV_100–340nm_ absorbance by seed coats, quercitrin flavonoid, and DNA, determined using synchrotron light and plotted with solar irradiance at 1 AU. (**A**) Ws wild type and *tt4-8* flavonoid minus. (**B**) Subtraction of *tt4-8* from Ws. (**C**) Quercitrin flavonoid. (**D**) Salmon sperm DNA. (**E**) Solar irradiance at 1 AU.

In an effort to characterize UV light penetration of Arabidopsis seed coats, UV_100–340nm_ absorption spectra (Fig. 5A) were compared for wild type Ws and mutant *tt4-8* seed coat fragments, prepared from dry Arabidopsis seeds and attached to MgF_2_ discs by their natural mucilage. These spectra have qualitative value, but possible differences in seed coat packing made quantitative results only approximate. Both the Ws wild type seed coats and the Arabidopsis flavonoid, quercitrin, showed absorption peaks at 200 nm (Fig. 5A, 5C). A second peak at 260 nm was recorded for quercitrin, with strongly increasing absorption for both Ws and quercitrin at wavelengths below 185 nm. In the Ws seed coats, the 260 nm peak was shifted to 277 nm. A subtraction of *tt4-8* from Ws gave a wavelength-dependent estimation of potential shielding by the seed coat flavonoids, with peaks at 200 and 277 nm (Fig. 5C). The seed coat absorption curves are aligned with DNA absorption and solar irradiance spectra for reference (Fig. 5D, 5E), showing vulnerability for DNA from UV_<200nm_. Solar irradiance decreases exponentially as wavelength shortens, while energy (not shown) increases in a linear fashion.

## 4. Discussion

Resistance to unprotected spaceflight is based on physical shielding from UV, repair of accumulated damage, and redundancy in essential functions. In the dry state, a seed's cellular contents are glass-like and nearly metabolically inert, with repair mechanisms quiescent (Bewley, 1997; Murthy *et al.,* 2003). Thus, free radicals and modifications in key entities, such as nucleic acids, membranes, and ribosomes, accumulate during exposure to space. When the seed imbibes water, metabolism resumes, repair begins, and toxic compounds are neutralized. If all goes well, the preformed root and shoot grow via cell enlargement and cell division, causing the root to emerge through the seed coat, providing the marker used here to define germination. Germination tests on a population of seeds thus reflect not only the ability to physically block incoming UV and cosmic radiation during exposure but also the ability to repair and circumvent accumulated damage upon germination. The kinetics and final level of germination provide global measures of the seeds' capacity to cope with the extreme environment of space.

Among the organisms tested in the EXPOSE-E missions, only plant seeds clearly survived full exposure in EXPOSE-E. In the second mission (EXPOSE-R), total UV doses were 1.4 times those of the EXPOSE-E experiment, and cosmic radiation doses were higher by a factor of 1.6. The lethality of solar UV was confirmed in EXPOSE-R in all species tested, including Arabidopsis seeds, whose germination in our experiments after return to Earth was reduced to a maximum of 3%. The 11 plants that developed did not survive transfer to soil (Fig. 1C), indicating that accumulated damage could not be overcome through repair and redundancy. In addition, Arabidopsis seeds were completely killed in a separate experiment on EXPOSE-R (Novikova *et al.,* 2015). A survival endpoint was thus reached in Arabidopsis, and the results from EXPOSE-E, where exposure was lower and better survival occurred, were corroborated and extended. The hypothesis drawn from EXPOSE-E, that Arabidopsis and tobacco seeds could have survived a direct, unprotected, transfer from Mars to Earth, remains a pertinent exercise in evaluating resistance against the scale of interplanetary distances.

The study of the effects of space exposure on the integrity of tobacco chloroplast DNA was motivated by the lack of morphological mutations in the survivors from EXPOSE-E, in contrast to a previous report (Kranz *et al.,* 1990). We found no differences in DNA integrity between the shielded sample in space (S2) and the shielded sample in the ground simulation (G2) (Fig. 2). However, compared to these dark layers, chloroplast DNA from seeds in the fully exposed space (S1) layer and in the ground simulation (G1) layer registered similar losses of integrity, measured by qPCR and marker rescue (Fig. 2). Conditions were the same in these exposed layers, except that the UV received by the ground simulation (Table 1) lacked the 110–200 nm region and cosmic radiation. Although it likely contributed, this lower part of the UV spectrum was thus not essential to the structural and functional damage recorded in the tobacco chloroplast DNA.

A comparison of germination results from the S1 and G1 samples gave an estimate of the effects of cosmic radiation plus UV_110–200nm_. This comparison was made in Arabidopsis seeds from EXPOSE-R, since the G1 results from EXPOSE-E were compromised by a mottled seed blackening artifact and a mismatch in the UV exposures (Table 1). Instead of the similarity in the S1 versus G1 comparison, seen in the DNA integrity experiments (Fig. 2), germination was robust in the ground simulation (Fig. 3B) but almost nonexistent in the S1 space exposure (Fig. 3D). The obvious explanation for the high germination in the G1 seeds is that they were spared exposure to UV_110–200nm_. However, the G1 seeds were also spared the cosmic radiation received by the S1 seeds. We can roughly estimate the effect of cosmic radiation in the S1 layer by examining the S2 layer (Fig. 3C), which received cosmic radiation but no UV light. Germination in the S2 layer was retarded and reduced in Ws and *tt4-8* and to a lesser extent in Col but not significantly in *fah1-2* (Fig. 3A, 3C). The S2 layer received about 75–85% of the radiation in the S1. Extrapolation to the S1 level does not explain the near kill of the S1 seeds. Corn seeds germinated after exposure to 500 krad of γ radiation (Schwartz and Bay, 1956), which is roughly 1000 times the cosmic radiation received in the S1 layer of EXPOSE-R. We conclude that exposure to UV_110–200nm_ caused the bulk of the near kill of the fully exposed Arabidopsis S1 seeds.

We can further define the part of the UV spectrum responsible for lethality, because the lower wavelengths (110–150 nm) were largely not transmitted in EXPOSE-R, according to transmission studies of pre- and postflight windows in the adjacent AMINO experiment (Demets *et al.,* 2015). Since UV_200–400nm_ was not lethal in the G1 layer (Fig. 3B), the majority of the deleterious UV was concentrated between 150 and 200 nm. This does not mean that UV_200–400nm_ is innocuous: a dose of 87 MJ m^−2^ of UV_254nm_ was sufficient to reduce germination in Arabidopsis Columbia seeds to 17% ± 3% (Fig. 4B). (Exposure of seeds behind Suprasil windows, which are not transparent to UV_<200nm_, was not possible in these missions, due to limitations in sample number, but Suprasil windows should be included in future experiments to confirm the deleterious role of the UV_110–200nm_ region.)

Both germination and chloroplast DNA integrity were low in the S1 layer; however, the correlation did not hold up for the G1 layer, which lacked UV_110–200nm_. Instead, germination in G1 was high, and DNA integrity was low (Figs. 2A, 2B, and 3B). However, DNA integrity was determined in tobacco from EXPOSE-E, while germination was measured in Arabidopsis from EXPOSE-R, where UV exposures and cosmic radiation were higher (Table 1). The importance of this experimental inconsistency can be discounted because higher exposure in the EXPOSE-R G1 would cause lower germination, not higher, and germination studies gave globally similar results in tobacco and Arabidopsis (Tepfer *et al.,* 2012). A more obvious explanation for the discrepancy between high germination and low DNA integrity in G1 is that the UV_110–200nm_ (lacking in G1) was not only making the major contribution to lethality in space but that this lethality was not due to the DNA damage. Rather, accumulated DNA damage was circumvented through redundancy and repair. This interpretation is in keeping with the lack of mutant phenotypes in EXPOSE-E (Fig. 1A).

Ribosomes are an example of targets potentially essential to the recovery process. UV-induced cross-linking between ribosomal RNA and proteins would inhibit protein synthesis and thus repair and recovery (Casati and Walbot, 2004). More general targets include substances with π electron systems: for example, nucleic acids, aromatic amino acids, flavonoids, and many secondary metabolites (*e.g.,* polyamines) (Zalar *et al.,* 2007a, 2007b). Incoming photons not stopped in the seed coat would damage (to give a few examples) chromatin, ribosomes, membranes (*e.g.,* via aromatic amino acids in transmembrane proteins), and enzymes (*e.g.,* via conserved aromatic amino acids in ligand binding sites). Arginine absorbs strongly at 178 nm and below (Zalar *et al.,* 2007b); thus, π electrons are not a requirement for absorption in this region. In summary, numerous substances absorb UV_110–200nm_, which is consistent with the observed lethality.

Absorption spectra (Fig. 5A, 5B), obtained through isolated seed coat fragments, showed that UV_100–340nm_ penetrates the seed coat and that the differences between the Ws wild type and the *tt4-8* mutant roughly correlate with the absorption spectrum of quercitrin, a principal seed coat flavonoid (Fig. 5A–5C). Since Arabidopsis seeds have only a vestigial endosperm, these penetrating photons would directly attack the embryo, where damage to the shoot and root meristematic regions will prevent the embryo from germinating. The shielding against UV_200–400nm_ by flavonoids was evident in the lack of germination of the *tt4-8* seeds from the G1 layer (Fig. 3B). Quercitrin flavonoid mimics UV_125–340nm_ absorption by salmon sperm DNA (Fig. 5C) (Zalar *et al.,* 2007a). Flavonoids exposed to space in EXPOSE-E lost structural features, but they retained their shielding functions (Tepfer *et al.,* 2012). They are concentrated in the seed coat, which is only loosely connected to the underlying embryo, as demonstrated by the method used here to prepare seed coat samples. In contrast, histones and ribosomal proteins bind to DNA in chromatin and absorb strongly throughout the region proposed here of particular sensitivity to UV (Zalar *et al.,* 2007b). Thus, UV energy absorbed by histones (and ribosomal proteins) would be transferred to associated nucleic acids through physical proximity. The seed embryo thus benefits from a physically distant, flavonoid-rich, protective shell provided by the mother plant. Flavonoids are also antioxidants that help cope with free radicals produced by energy absorbed by more general targets than those discussed here. We proposed that contemporary flavonoid synthesis functions were acquired from the original cyanobacterial endosymbiont and later lost in free-living cyanobacteria after the accumulation of UV-shielding oxygen in the atmosphere (Zalar *et al.,* 2007a).

Germination of morning glory seeds after exposure to germicidal UV_254nm_ was dose-dependent. Arabidopsis and tobacco seeds were completely killed at the maximum dose, which was 2.4 times the UV_110–400nm_ that killed Arabidopsis seeds in EXPOSE-R. Although UV_254nm_ is not a substitute for exposure to the full spectrum of solar light under space conditions, the complete resistance of morning glory seeds indicates that solar UV would be far less detrimental to morning glory than to Arabidopsis or tobacco seeds. Larger and more resistant seeds should be included in future experiments in space.

## Abbreviations Used

ISS: International Space Station
PCR: polymerase chain reaction
qPCR: quantitative polymerase chain reaction
